# Antimicrobial Resistance Pattern of *Escherichia coli* Isolated from Frozen Chicken Meat in Bangladesh

**DOI:** 10.3390/pathogens9060420

**Published:** 2020-05-28

**Authors:** Mst. Sonia Parvin, Sudipta Talukder, Md. Yamin Ali, Emdadul Haque Chowdhury, Md. Tanvir Rahman, Md. Taohidul Islam

**Affiliations:** 1 Population Medicine and AMR Laboratory, Department of Medicine, Faculty of Veterinary Science, Bangladesh Agricultural University, Mymensingh 2202, Bangladesh; soniaparvin@bau.edu.bd (M.S.P.); s.talukder39857@bau.edu.bd (S.T.); yamin2301@gmail.com (M.Y.A.); 2 Department of Pathology, Faculty of Veterinary Science, Bangladesh Agricultural University, Mymensingh 2202, Bangladesh; emdad001@yahoo.com; 3 Department of Microbiology and Hygiene, Faculty of Veterinary Science, Bangladesh Agricultural University, Mymensingh 2202, Bangladesh; tanvirahman@bau.edu.bd

**Keywords:** *Escherichia coli*, antimicrobial resistance, ESBL, MDR, frozen chicken meat, Bangladesh

## Abstract

*Escherichia coli* is known as one of the most important foodborne pathogens in humans, and contaminated chicken meat is an important source of foodborne infection with this bacterium. The occurrence of extended-spectrum β-lactamase (ESBL)-producing *E. coli* (ESBL-Ec), in particular, in chicken meat is considered a global health problem. This study aimed to determine the magnitude of *E. coli*, with special emphasis on ESBL-Ec, along with their phenotypic antimicrobial resistance pattern in frozen chicken meat. The study also focused on the determination of ESBL-encoding genes in *E. coli*. A total of 113 frozen chicken meat samples were purchased from 40 outlets of nine branded supershops in five megacities in Bangladesh. Isolation and identification of *E. coli* were done based on cultural and biochemical properties, as well as PCR assay. The resistance pattern was determined by the disc diffusion method. ESBL-encoding genes were determined by multiplex PCR. The results showed that 76.1% of samples were positive for *E. coli*, of which 86% were ESBL producers. All the isolates were multidrug-resistant (MDR). Resistance to 9–11 and 12–13 antimicrobial classes was observed in 38.4% and 17.4% isolates, respectively, while only 11.6% were resistant to 3–5 classes. Possible extensive drug resistance (pXDR) was found in 2.3% of isolates. High single resistance was observed for oxytetracycline (93%) and amoxicillin (91.9%), followed by ampicillin (89.5%), trimethoprim–sulfamethoxazole, and pefloxacin (88.4%), and tetracycline (84.9%). Most importantly, 89.6% of isolates were resistant to carbapenems. All the isolates were positive for the *bla*TEM gene. However, the *bla*SHV and *bla*CTX-M-2 genes were identified in two ESBL-non producer isolates. None of the isolates carried the *bla*CTX-M-1 gene. This study provided evidence of the existence of MDR and pXDR ESBL-Ec in frozen chicken meat in Bangladesh, which may pose a risk to human health if the meat is not properly cooked or pickled raw only. This emphasizes the importance of the implementation of good slaughtering and processing practices by the processors.

## 1. Introduction

*Escherichia coli* (*E. coli*), a member of the *Enterobacteriaceae* family, is a normal inhabitant of the gut of poultry and a frequent microbial contaminant of retail poultry meat [1]. *E. coli* is also known as one of the most important foodborne pathogens in humans, which may be associated with a diversity of acute and invasive infections in humans, and it can easily be disseminated in different ecosystems through the food chain [2,3]. It is a highly versatile bacterial species comprising both nonpathogenic strains and different pathogenic variants with the ability to cause either intestinal or extra-intestinal diseases [4]. While the majority of strains of *E. coli* are nonpathogenic in humans (e.g., uncomplicated urinary tract infections) or exist as part of the indigenous flora, often contributing to the vital tasks performed by the intestinal microflora, some strains of poultry-derived *E. coli* can also be opportunistic and pathogenic in nature (e.g., bloodstream infections) [4]. Chicken meat is frequently contaminated by *E. coli* during handling, improper dressing, cleaning, and unhygienic practices of selling meat. Contaminated chicken meat is considered as a potential source of infection with *E. coli*, either via direct contact during food preparation or via consumption of undercooked or raw meat products [3]. Although *E. coli* exhibits heat sensitivity to thermal treatment ranging from 60 to 80 °C, some strains of *E. coli* were reported to be highly resistant to heat [5]. It was reported that several strains of *E. coli* become resistant to heat by the addition of salt, and about 2% of *E. coli*-including food isolates harbor heat-resistant genes and show increased heat resistance [6]. The locus of heat resistance (LHR) can be transferred to another *E. coli* through lateral gene transfer [7]. Moreover, the duration of microwave exposure and the methods used for cooking can also result in failure of the thermal inactivation of *E. coli* [8]. *E. coli* can also survive low-temperature stress (cold shock) through different mechanisms. The synthesis of cold-shock proteins (CSPs) is one of the most important responses to cold temperature, and these are involved in a variety of essential functions such as transcription, translation, mRNA degradation, protein synthesis, and recombination in *E. coli* [9,10]. However, it was reported that rapid chilling (2000 °C∙min^−1^) induces an immediate loss of viability of up to more than 90% for exponentially growing cells of *E. coli* [11]. The most common symptoms of food poisoning due to *E. coli* are abdominal cramps, vomiting, and, in some cases, bloody diarrhea in humans. Sometimes, the infection caused by *Shiga* toxin-producing *E. coli* may lead to hemolytic–uremic syndrome that can cause kidney failure [12]. In most of the cases, *E. coli* infections are self-limiting, and antibiotic medication is discouraged [13].

Over the past few decades, antibiotic resistance trends increased at a faster rate among chicken isolates of *E. coli* than human clinical isolates [14]. Commensal *E. coli* were determined as an important reservoir of antimicrobial resistance genes that may spread to pathogenic strains [15]. One of the most common resistance mechanisms reported in the members of the family *Enterobacteriaceae* is the production of β-lactamase enzymes that hydrolyze β-lactam antibiotics [16]. Extended-spectrum β-lactamases (ESBLs), variants of β-lactamases, a heterogeneous group of enzymes, are encoded by genes which efficiently hydrolyze third- and fourth-generation cephalosporins and monobactams (e.g., aztreonam) but are inhibited by β-lactamase inhibitors such as clavulanic acid and tazobactam [17]. *E. coli* that produce ESBL are of particular concern because of the implications for human and food animal health worldwide [18]. The emergence of ESBLs is considered an important cause of transferable multidrug-resistant superbugs, particularly *E. coli*. Furthermore, ESBL-producing *E. coli* often exhibit co-resistance to multiple classes of antimicrobials, mainly fluoroquinolones, sulfonamides, aminoglycosides, chloramphenicol, trimethoprim, and tetracyclines, which may increase the risk of poor clinical outcomes due to lack of effective treatment options [18].

The major genes responsible for ESBL production include TEM genes (*bla*TEM), SHV genes (*bla*SHV), and CTX-M genes (*bla*CTX-M). The CTX-M type ESBL-producing *E. coli* is the most dominant globally [19]. In Bangladesh, *bla*CTX-M-1 (94.4%) and *bla*TEM (50–91.3%) ESBL-producing *E. coli* were reported in droppings of chickens [20,21,22]. Chickens are considered as a potential reservoir of ESBL-producing *E. coli* [23]. Chicken meat contaminated with ESBL-producing bacteria is thought to be one of the potential risk factors for the wide dissemination of ESBL-producing bacteria in humans [24].

The prevalence of multidrug-resistant superbugs and ESBL-producing bacteria is increasing in humans, as well as animals. Multidrug-resistant (MDR) and extensively drug-resistant (XDR) bacteria are well-defined by the European Center for Disease Control, and Centers for Disease Control and Prevention, Atlanta [25]. MDR is defined as acquired non-susceptibility to at least one agent in three or more antimicrobial categories, and XDR is defined as non-susceptibility to at least one agent in all but two or fewer antimicrobial categories (i.e., bacterial isolates remain susceptible to only one or two antimicrobial categories).

Currently, like in other countries, the lifestyle, preference, and demands of consumers in Bangladesh are changing rapidly. With the current shopping practice, supershops are now a necessity as they offer a unique shopping experience with all essential commodities under one roof. Consumers, especially city dwellers, are increasingly becoming more aware of their convenience and the lifestyle they allow, as they prefer to go to supershops rather than to wet markets to buy their everyday stuff, including frozen chicken meat. City dwellers tend to buy frozen chicken meat along with other frozen and ready-to-cook foodstuffs as these frozen items need minimal processing for cooking and, thus, they can save time [26]. However, the microbiological safety of this frozen chicken meat is an important concern in the context of public health hazards, as two studies reported bacterial contamination in frozen chicken meat in Dhaka city of Bangladesh [27,28]. Both studies were restricted to three to five supershops of Dhaka city only. Furthermore, none of these two reports investigated the multidrug resistance pattern of ESBL-producing *E. coli*. Therefore, a study is required to have an updated scenario of *E. coli* contamination along with the resistance pattern in frozen chicken meat covering more outlets of available branded supershops located in five megacities of Bangladesh. The present study determined the (i) prevalence and distribution of *E. coli*, with special emphasis on ESBL-producing *E. coli*, along with their phenotypic resistance pattern, in frozen chicken meat sold in various supershops located in five megacities of Bangladesh, and (ii) ESBL-encoding genes in *E. coli* in frozen chicken meat, which are yet to be investigated in Bangladesh.

## 2. Results

### 2.1. Source of Chicken, and Processing and Packaging of Frozen Chicken Meat

The findings of the questionnaire survey, conducted in 40 outlets of nine branded supershops of five megacities in Bangladesh, revealed that supershops of all brands purchased chickens from their contract farms (Table 1). All the outlets of brands 4, 6, 8, and 9, and majority outlets of brands 1 to 3 had their chicken meat processed outside the supershops. Regarding the packaging of meat, it was observed that 100% of outlets of brands 6, 8, and 9, and the majority of brands 1 to 3 packaged their chicken meat inside the shops. However, all outlets of brand 5 processed and packaged chicken meat inside the shop; in contrast, all outlets of brand 7 did it outside the shop.

### 2.2. Prevalence and Distribution of ESBL-Producing and ESBL-Non-Producing E. coli

**Figure 1:**
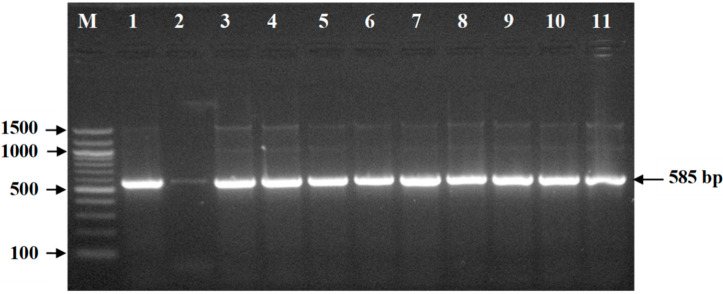
PCR amplified product of 585 bp from *16S rRNA* gene of *E. coli* following 1.5% agarose gel electrophoresis and ethidium bromide staining. Legends: M = DNA marker (100 bp), Lane 1 = Positive control of *E. coli*, Lane 2 = Negative control, Lanes 3–11 = PCR product of tested *E. coli* isolates.

The overall prevalence of *E. coli* was 76.1% (86/113) in frozen chicken meat samples, and it varied from 33.3% to 100% among the nine different brands (Table 2). All *E. coli* isolates were confirmed by PCR as they generated a 585-bp fragment size on amplification (Figure 1). Out of 86 *E. coli* isolates, 74 (86%) were ESBL–producing *E. coli* (ESBL-Ec) and 14% (12/86) were ESBL-non-producing *E. coli* (non-ESBL-Ec) (Table 2). None of the *E. coli* isolates were recovered from one brand (brand 9). The prevalence of ESBL-Ec and non-ESBL-Ec varied significantly from brand to brand. The prevalence of ESBL-Ec in frozen chicken meat of different brands varied from 50% to 100%, while the prevalence of non-ESBL-Ec varied from 30% to 100% (Table 2).

As shown in Table 3, the highest prevalence of ESBL-Ec was recorded in both Chattogram and Mymensingh divisions (100.0%), followed by the Dhaka (92.3%) division, which was significantly higher than that in the Rajshahi division (33.3%). On the other hand, the highest prevalence of non-ESBL-Ec was in the Sylhet (100.0%) division and the lowest was in the Dhaka division (7.7%). Moreover, in broiler and cockerel chickens, similar prevalence of ESBL-Ec (87.3% and 82.6%, respectively) and non-ESBL-Ec (12.7% and 17.4%, respectively) was observed (Table 3). We did not find any significant differences in the prevalence of both ESBL-Ec and non-ESBL-Ec between organic and non-organic chickens. Considering the types of meat sample, although the highest isolation rate of ESBL-Ec was found in leg muscle (100%) there were no significant differences between different types of meat sample. The isolation rate of non-ESBL-Ec was highest in breast muscle (18.2%) and lowest in drumstick (9.1%) (Table 3).

### 2.3. Distribution of Possible Extensively Drug-Resistant (pXDR) E. coli

Notably, in this study, 2.3% (2/86) of *E. coli* isolates were pXDR (resistant to 13 out of 16 antimicrobial classes). The pXDR *E. coli* isolates were only susceptible to polymyxin, monobactam, and glycylcycline antimicrobial classes. One pXDR *E. coli* isolate was recovered from broiler meat of brand 5 in Dhaka division and another one was recovered from the cockerel meat of brand 7 in Mymensingh division. Both pXDR isolates originated from non-organically produced chickens.

### 2.4. Distribution of Multidrug-Resistant E. coli

**Figure 2:**
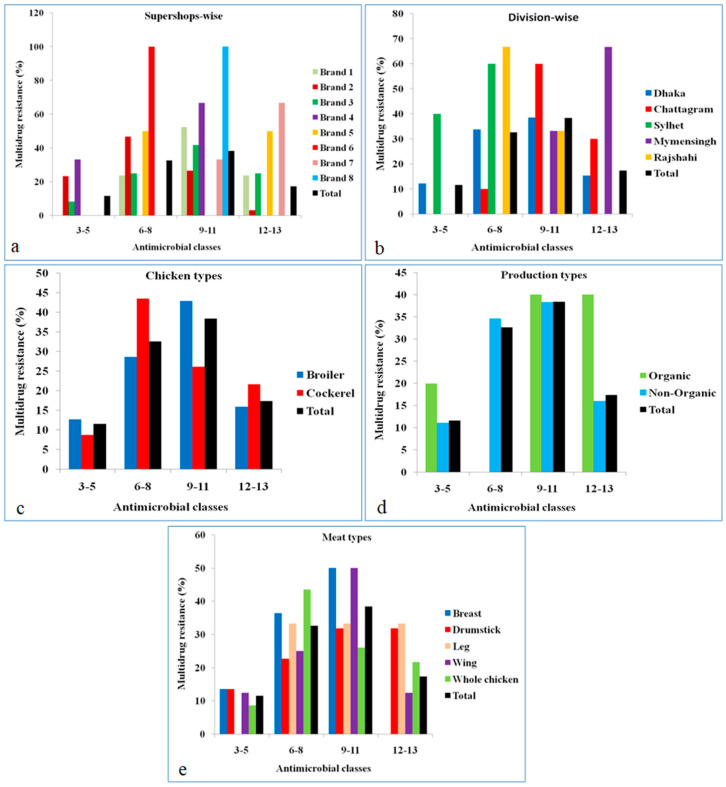
(**a**–**e**) Antimicrobial class-wise distribution of multidrug resistance pattern of *E. coli* isolated from frozen chicken meat.

Of the 86 *E. coli* isolates tested, all the isolates were multidrug-resistant (MDR). In our study, we used 16 antimicrobial classes. The overall distributions of MDR *E. coli* are shown in Figure 2a–e. It was observed that 38.4% of isolates were resistant to 9–11 antimicrobial classes, 32.6% were resistant to 6–8 classes, and 11.6% were resistant to 3–5 classes. Notably, 17.4% of isolates were resistant to 12–13 antimicrobial classes. Multidrug-resistant *E. coli* were widespread among different brands, and all isolates from brand 6 and brand 8 showed a higher rate of resistance to 6–8 and 9–11 antimicrobial classes, respectively (Figure 2a). Regarding the division-wise distribution of MDR *E. coli*, the highest percentage of isolates, resistant to 6–8 and 12–13 antimicrobial classes, was observed in Rajshahi and Mymensingh divisions, respectively (Figure 2b). Considering chicken types, it was revealed that 43.5% of isolates from cockerel chicken meat, and 42.9% of isolates from broiler chicken meat were resistant to 6–8 and 9–11 antimicrobial classes, respectively (Figure 2c). Production type-wise MDR pattern results revealed that 40% of isolates from organically produced chickens were resistant to 9–11 and 12–13 antimicrobial classes, respectively, while 38.3% of isolates from non-organically produced chickens were resistant to 9–11 antimicrobial classes (Figure 2d). Looking at the meat sample type-wise distribution, 50% of the isolates, recovered from breast and wing muscle, were resistant to 9–11 antimicrobial classes (Figure 2e).

It is noted that, among the 86 *E. coli* isolates, all isolates were resistant to at least four, and up to 28 antimicrobials (Table 4). The frequency of resistance to 19–23 antimicrobials was observed in 22 (25.6%) isolates, while only 11 (12.8%) isolates were resistant to 4–8 antimicrobials. The percentage of resistance to 9–13 and 14–18 antimicrobials was the same (22.1%). Notably, 15 (17.4%) isolates were resistant to 24–28 antimicrobials. Most importantly, resistance to three or fewer antimicrobials was not observed in any of the isolates tested. Brand-wise resistance to antimicrobials revealed that the highest resistance to 19–23 antimicrobials was observed in 42.9% isolates from brand 1. Two (66.7%) isolates from brand 7, one (50%) from brand 5, and 7 (29.2%) from brand 3 were resistant to 24–28 antimicrobials (Table 4). Significant differences were observed in the resistance percentages to antimicrobial agents between brands.

**Figure 3:**
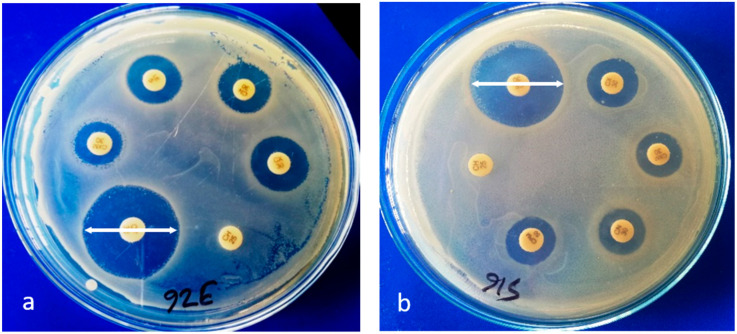
(**a**,**b**) Antimicrobial susceptibility tests of *E. coli* by disc diffusion method showing zone of inhibition (↔).

Overall analysis of disc diffusion results (Figure 3a,b) revealed that the highest single resistance in *E. coli* was detected against oxytetracycline (93%) and amoxicillin (91.9%). In addition, resistances to ampicillin (89.5%), trimethoprim–sulfamethoxazole and pefloxacin (88.4%), tetracycline (84.9%), cefepime (72.1%), and piperacillin–tazobactam (70.9%) were also very high in *E. coli* isolates (Table S1, Supplementary Materials). Among all the antibiotics, resistance to aztreonam was observed to be the lowest (1.2%), followed by ceftriaxone and tigecycline (2.3%) (Table S1, Supplementary Materials).

The variation in the resistance pattern of ESBL-Ec (*n* = 74) and non-ESBL-Ec (*n* = 12) isolates was determined (Table S1, Supplementary Materials). Resistances to oxytetracycline and amoxicillin (91.9%), ampicillin and trimethoprim–sulfamethoxazole (89.2%), pefloxacin (87.8%), cefepime (81.1%), piperacillin–tazobactam (73.0%), and doxycycline (70.3%) were found to be higher in ESBL-Ec isolates, while resistances to oxytetracycline (100.0%), tetracycline, pefloxacin, ampicillin, and amoxicillin (91.7%), and trimethoprim–sulfamethoxazole (83.3%) were observed to be higher in non-ESBL-Ec isolates. No significant differences were observed among these antimicrobial agents between ESBL-Ec and non-ESBL-Ec except for cefepime, streptomycin, and chloramphenicol. It is important to note that 77 (89.5%) isolates showed resistance to carbapenems, the antimicrobials used in human medicine, of which 76 isolates were ESBL-Ec. The resistance to imipenem was 47.7%, and that to meropenem was 41.9%.

### 2.5. Genotypes of ESBL-Ec and Non-ESBL-Ec

**Figure 4:**
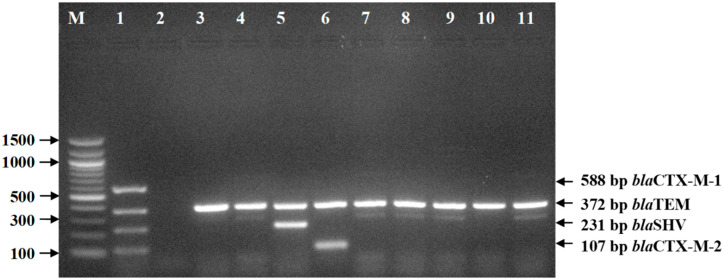
ESBL-encoding genes of *E. coli* isolates from frozen chicken meat by multiplex PCR, followed by 1.5% agarose gel electrophoresis and ethidium bromide staining. Legends: M = DNA marker (100 bp), Lane 1 = Positive control, Lane 2 = Negative control, Lanes 3–11 = Positive for *bla*TEM gene; Lane 5 = Positive for *bla*SHV gene; Lane 6 = Positive for *bla*CTX-M-2 gene.

The findings of ESBL genes, i.e., *bla*TEM, *bla*SHV, *bla*CTX-M-1, and *bla*CTX-M-2 genes, are presented in Table 5 and Figure 4. All the isolates were positive for the *bla*TEM gene. One isolate of non-ESBL-Ec was positive for the *bla*SHV gene, and another one isolate of non-ESBL-Ec was positive for the *bla*CTX-M-2 gene. None of the tested isolates harbored the *bla*CTX-M-1 gene.

## 3. Discussion

*E. coli* is a common enteric pathogen, specific strains of which can cause human and animal disease. It is one of the groups of seven species that the World Health Organization (WHO) highlighted as of key antimicrobial resistance (AMR) concern, and it serves as a sentinel organism for the assessment of the development of antimicrobial resistance [29]. The emergence and spread of ESBL-Ec linked to chickens and other farm animals are of particular concern [23].

The present study reports the first comprehensive findings on the extent and distribution of ESBL-Ec and their antimicrobial resistance pattern including resistance genes in frozen chicken meat collected from almost all branded supershops located in five megacities of Bangladesh. This study showed the high prevalence (76.1%) of *E. coli* in frozen chicken meat compared with 49–53% prevalence in raw chicken meat as reported earlier in Bangladesh [30,31], 66.3% in India [32], 47.1% in Nepal [33], and 50.5% in Korea [34], and this may be a potential hazard to the consumers. The difference in the prevalence of *E. coli* may be attributed to several factors including the source of meat, sample number, isolation methods, possible cross-contamination during slaughtering, slaughterhouse sanitation, and personal hygiene, as well as other practices through to the food chain.

One of the main findings in this study was the high prevalence (86.0%) of ESBL-Ec in frozen chicken meat. These results corroborate the findings of similar studies conducted in Japan, in which the authors reported that 65–77% of frozen chicken meat samples were contaminated with ESBL-Ec [35,36]. The present study observed that the prevalence of ESBL-Ec in frozen chicken meat varied from brand to brand, which might be due to variation in processing, packaging, and personnel hygienic practices in different supershops. It is expected that different brands follow different types of management and, thus, there are different risks regarding the prevalence of ESBL-Ec. Contamination may also occur during the transportation of chicken meat from farm to supershops or during the steps involved in slaughtering, defeathering, plucking, and chilling of the chicken meat [37]. The distribution of ESBL-Ec was found to vary from division to division, with Chattogram, Mymensingh, and Dhaka divisions having the highest prevalence and the Rajshahi division having the lowest prevalence. The highest distribution of non-ESBL-Ec was observed in the Sylhet division of Bangladesh. An earlier study showed that 30% of ESBL-Ec was detected from droppings of domestic chickens in the Rajshahi division of Bangladesh [38]. In the present study, a considerably high percentage of ESBL-Ec was recovered from different types of meat samples. The pathogenic *E. coli* are usually absent in the muscle tissue and body fluids of healthy living chickens, but they can enter into the meat during slaughtering or at the time of processing and packaging from the gastrointestinal tract [39]. This high prevalence is very alarming and requires risk assessments and pertinent risk management to keep down the occurrence and spread of ESBL-Ec. This result also indicates that the contamination of frozen chicken meat with ESBL-Ec in Bangladesh is more frequent, which may rapidly raise the risk of humans being infected.

It is of particular concern that all the isolates of *E. coli* in this study were MDR, of which a substantial percentage of isolates showed resistance to 9–13 classes of antimicrobials, which is in line with previous observations among *E. coli* recovered from retail chicken meat in Korea [34], but which differed from some other reports [31,33]. The highest percentage of isolates from Rajshahi and Mymensingh divisions expressed MDR, which is in disagreement with previous reports in Bangladesh, in which 10–35% of *E. coli* isolates in retail chicken meat from Mymensingh and Dhaka divisions showed MDR [40,41]. Of note, the current study also observed that 2.3% of *E. coli* isolates were pXDR. An earlier report from Japan detected extensive MDR *E. coli* in 70% of frozen chicken meat samples [36]. The high rates of MDR and existence of pXDR in this study imply that this can reflect the frequent use or misuse of antimicrobials along with poor biosecurity and waste management systems in poultry production in Bangladesh, which creates a selection pressure, thus contributing to the emergence and spread of MDR bacteria in poultry production systems. Indeed, MDR in commensal bacteria develops naturally over time, usually through genetic changes and/or via the action of MDR efflux pumps; however, the massive use of antimicrobial agents for disease control and prevention causes an unprecedented increase in resistance [42,43]. Moreover, the use of disinfectants, particularly quaternary ammonium compounds (QACs), to limit infection in poultry may also induce the AMR through cross-resistance between QACs and a range of antimicrobials [44,45]. Another plausible explanation is that the high prevalence of MDR *E. coli* may be attributed to the possible cross-contamination during slaughtering, cutting, and further processing indirectly through contaminated equipment, as well as the use of stored water in containers that received minimal cleaning after frequent washing of carcasses [37]. These observations support the possibility that chicken meat might be one of the potential sources of MDR *E. coli* infections causing possible transmission of resistant bacteria to consumers, and they suggest that continued surveillance is important.

Increasing rates of antimicrobial resistance in both ESBL-Ec and non-ESBL-Ec are a growing public health problem that needs to be monitored continuously. Our study indicated that all isolates of *E. coli* exhibited absolute resistance (100%) to at least four antimicrobial agents. Of note, 17.4% isolates of *E. coli* showed resistance to more than 24 antimicrobials. A high percentage of antimicrobial-resistant *E. coli* from frozen chicken meat was also reported by several investigators [33,36]. In the current study, oxytetracycline resistance was the most frequently observed antimicrobial resistance in both ESBL-Ec and non-ESBL-Ec, which is consistent with several other studies in frozen chicken meat [33,46]. The finding is not surprising because, since its approval in 1948, oxytetracycline was widely used in veterinary practices, which probably led to this outcome [47].

A very high degree of resistance was also observed for amoxicillin, ampicillin, and trimethoprim–sulfamethoxazole in both ESBL-Ec and non-ESBL-Ec. A similar resistivity pattern was observed in Bangladesh [31,41], Japan [35], Korea [34], and Vietnam [48]. This may be attributed to the long-term and indiscriminate use of these antimicrobial agents in poultry production in Bangladesh [20]. As fluoroquinolones and cephalosporins are the drugs of choice for the treatment of bacterial infection in humans, *E. coli* resistant to these antimicrobials could represent a big challenge to animal and human therapeutic interventions, becoming a symbol a relevant public health implication [49]. Unfortunately, this study demonstrated that the prevalence of fluoroquinolone (mainly pefloxacin) resistance in both ESBL-Ec and non-ESBL-Ec was also very high. This result may imply the more frequent use of fluoroquinolones in poultry production in Bangladesh. Moreover, more than 80% isolates of ESBL-Ec showed resistance to cefepime, a fourth-generation cephalosporin antimicrobial, which is higher than a previous observation in retail chicken meat (4.8%) in Korea [34]. Cephalosporin resistance is a matter of concern because cefepime is not used in veterinary practices in Bangladesh, and it is worrisome to find these phenotypes in chicken meat. The rate of resistance to multiple antimicrobials among ESBL-Ec isolates is usually common due to the carrying of multi-resistance genes and plasmids [50]. These plasmids can also carry genes for co-resistance to multiple classes of antimicrobials including fluoroquinolones, sulfonamides, aminoglycosides, chloramphenicol, trimethoprim, and tetracyclines [18]. Surprisingly, remarkably high resistance prevalence was found against carbapenems (last-line therapeutics to treat multidrug-resistant superbugs), mainly imipenem and meropenem, although carbapenems are not used in poultry practices in Bangladesh. There was no clear explanation for these high levels of resistance, but it might be due to co-selection and/or cross-resistance generated by other antimicrobials [51].

On the other hand, a relatively low resistance rate to aztreonam, ceftriaxone, and tigecycline was observed, probably because these antimicrobials are not used in poultry practices in Bangladesh, resulting in a lack of selective pressure by these antimicrobials in poultry production. It also supports the contention that antimicrobial resistance, induced once, is difficult to eliminate, because of associated resistance to other related antimicrobials [52]. Therefore, resistance to these antimicrobials should be carefully monitored.

Among the prevalent ESBL-Ec genes from chicken meat, *bla*TEM, *bla*SHV, and *bla*CTX-M (*bla*CTX-M-1 and *bla*CTX-M-2) are considered to be most diverse. The ESBL genes are usually located on plasmids, which could promote the dissemination of ESBL genes in Gram-negative bacteria [23]. The most prevalent ESBL-encoding gene in the current study was *bla*TEM, which is consistent with a similar study conducted in Vietnam [48]. Interestingly, *bla*SHV and *bla*CTX-M-2 were detected in two non-ESBL-Ec isolates. No *bla*CTX-M-1 was detected in this study. These findings are inconsistent with earlier studies in Bangladesh, where more than 50% of *E. coli* isolates from droppings of chickens harbored the *bla*TEM gene and 94.4% carried the *bla*CTX-M-1 gene [21,38]. It may be hypothesized that frozen chicken meat which is sold to the consumers could potentially act as a major source of gut colonization by avian strains of *E. coli* that carry *bla*TEM ESBL genes. 

It would be worthwhile if we could sample more outlets of supershops. However, frozen chicken meat samples were purchased from 40 outlets of almost all the renowned branded supershops located in five megacities of Bangladesh; thus, the data represent the scenario of all Bangladesh. This study seems to indicate the current status of contamination with ESBL-Ec in frozen chicken meat. It would be very important to investigate horizontal gene transfer, such as exchanges of plasmid or mobile genetic elements carrying genes for ESBLs, between bacteria isolated from chicken meat.

## 4. Materials and Methods

### 4.1. Sample Collection

**Figure 5:**
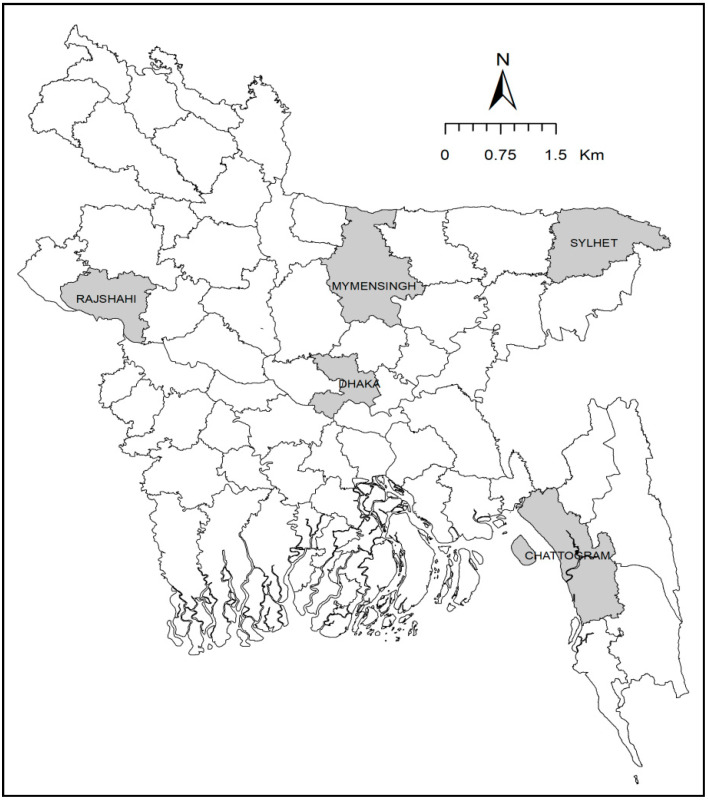
Map showing sampling sites in five megacities of Bangladesh.

A cross-sectional survey was conducted in 40 supershop outlets of nine brands available in five megacities (Dhaka, Sylhet, Mymensingh, Chattogram, and Rajshahi) of Bangladesh (Figure 5) from April to December 2019. A total of 113 frozen chicken meat samples (82 broiler chicken meat, 31 cockerel chicken meat) were purchased from these outlets. On availability, meat samples included whole chicken or chopped chicken comprising breast, drumstick, leg, and wing muscle. Samples were placed in separate sterile plastic bags, labeled, kept in an icebox, and transported to the laboratory and processed as soon as possible. Simultaneously, data on the brand name, source of chicken, processing and packaging of meat, and special labels (green chicken/organic) were collected.

### 4.2. Enrichment and Identification of E. coli

The preparation of the meat samples was based on the European standard ISO-16654:2001 [53]. During processing, frozen chicken meat was kept at room temperature until thawing; then, the meat surface was sterilized by stabbing with a hot spatula and the upper portion of meat was removed carefully. Then, about 25 g of the meat samples were chopped into very small fine pieces using sterile scissors and a scalpel, mixed with 225 mL of buffered peptone water, homogenized for two minutes with gentle shaking, and enriched overnight (18–24 h) at 37 °C. After pre-enrichment, 1 mL of diluted meat samples were taken using a sterile pipette and transferred into a test tube containing the nutrient broth and incubated overnight at 37 °C. Then, a loopful of this overnight culture was streaked onto Eosin Methylene Blue agar in duplicate and incubated at 37 °C for 18–24 h. Three presumptive *E. coli* colonies having a dark blue color with a characteristic metallic sheen from each selective agar plate were picked and then subcultured to obtain a pure culture, and identification was performed using standard microbiological and biochemical procedures including Gram staining, catalase, oxidase, indole, methyl red, Voges–Proskauer tests, and a sugar fermentation test using triple sugar iron agar. Positive isolates were stored in nutrient broth containing 50% (*v*/*v*) glycerol at −20 °C for further study.

### 4.3. Molecular Detection of E. coli

Bacterial DNA was extracted by boiling of 1 mL of overnight culture as described earlier [54]. The DNA concentration was measured by NanoDrop One (Thermo Fisher Scientific, Waltham, Massachusetts, USA). PCR was performed for the confirmation of *E. coli* using *16S rRNA* gene-specific primers as described earlier [55]. The sequence of the forward primer was 5′–GACCTCGGTTTAGTTCACAGA–3 and that of the reverse primer was 5′–CACACGCTGACGCTGACCA–3′. Amplification reactions were done in a 25-μL volume containing 12.5 μL of PCR Master Mix (Thermo Scientific, Waltham, Massachusetts, USA), 1.5 μL (15 pmol) of each forward and reverse primer, 0.5 μL of template DNA (50 ng), and 9.0 μL of nuclease-free water. The PCR was run under the following conditions in a Veriti™ 96-Well Thermal Cycler (Thermo Fisher Scientific Inc., Waltham, Massachusetts, USA): initial denaturation at 95 °C for 5 min followed by 35 cycles of amplification, denaturation for 1 min at 94 °C, annealing at 58°C for 1 min, extension for 1 min at 72 °C, and final extension at 72 °C for 7 min. After amplification, the PCR product was subjected to electrophoresis on 1.5% agarose gel containing ethidium bromide (5µg/mL). The resulting band of the PCR product was examined under an ultraviolet (UV) transilluminator and documented.

### 4.4. Antimicrobial Susceptibility Testing

Antimicrobial susceptibility was determined by disc diffusion assay with 38 antimicrobials belonging to16 antimicrobial classes. The following antimicrobial discs (Biomaxima, Lublin, Lubelskie, Poland; Oxoid, Basingstoke, Hampshire, UK) were procured and used for the testing:

(A) Non-extended spectrum cephalosporins including
  - First-generation cephalosporins: cephalexin (30 µg), cefradine (30 µg);
  - Second-generation cephalosporins: cefuroxime (30 µg), cefaclor (30 µg);
(B) Extended-spectrum cephalosporins including
  - Third-generation cephalosporins: cefotaxime (30 µg), ceftriaxone (30 µg), ceftazidime (30 µg), cefixime (5 µg);
  - Fourth-generation cephalosporins: cefepime (30 µg);
(C) Cephamycins: cefoxitin (30 µg);
(D) Fluoroquinolones: nalidixic acid (30 µg), ciprofloxacin (5 µg), levofloxacin (5 µg), norfloxacin (10 µg), ofloxacin (5 µg), gatifloxacin (5 µg), pefloxacin (5 µg);
(E) Penicillins: ampicillin (10 µg), amoxycillin (10 µg);
(F) Penicillins + β-lactamase inhibitors: amoxicillin–clavulanic acid (30 µg);
(G) Antipseudomonal penicillins + β-lactamase inhibitors: pipercillin–tazobactam (110 µg);
(H) Carbapenems: imipenem (10 µg), meropenem (10 µg);
(I) Polymyxins: colistin (10 µg), polymyxin B (300 units);
(J) Monobactams: aztreonam (30 µg);
(K) Aminoglycosides: gentamicin (10 µg), amikacin (30 µg), streptomycin (10 µg), neomycin (30 µg), tobramycin (10 µg);
(L) Tetracyclines: tetracycline (30 µg), oxytetracycline (30 µg), doxycycline (10 µg);
(M) Folate pathway inhibitors: trimethoprim–sulfamethoxazole (25 µg);
(N) Glycylcyclines: tigecycline (15 µg);
(O) Phenicols: chloramphenicol (30 µg);
(P) Macrolides: azithromycin (15 µg).

After preparation of each bacterial suspension, the turbidity was adjusted equivalent to 0.5 McFarland standard and then inoculated onto Mueller–Hinton agar in duplicate. After overnight incubation at 37 °C, the diameter of the clear zone of inhibition around each antimicrobial disc was measured in millimeters. These results were interpreted as per the guidelines of the Clinical and Laboratory Standards Institute (CLSI) [56]. The isolates were classified as susceptible, intermediate, and resistant. Isolates resistant to ≥ 1 agent in three or more antimicrobial classes were classed as multidrug-resistant (MDR), and isolates resistant to ≥ 1 agent in all but ≤ 2 antimicrobial classes were categorized as extensively drug resistant (XDR) [25].

### 4.5. Detection of ESBL-Producing E. coli

ESBL-producing *E. coli* were detected by a double-disc synergy technique, in which an amoxicillin/clavulanic acid disc (amoxicillin 20 µg and clavulanic acid 10 µg) was placed in the center of a plate, and cefotaxime (30 µg), ceftazidime (30 µg), and ceftriaxone (30 µg) discs were placed 30 mm (center to center) apart from the amoxicillin/clavulanic acid disc. The enhancement of the zone of inhibition of any one of the three discs toward the disc containing clavulanic acid suggested the presence of extended-spectrum β-lactamases [57]. The isolates that produced a zone of inhibition ≥ 22 mm for ceftazidime, ≥27 mm for cefotaxime, and ≥25 mm for ceftriaxone were considered as potential ESBL producers as recommended by CLSI [56].

### 4.6. Detection of ESBL-Encoding Genes

The ESBL-encoding genes (*bla*TEM, *bla*SHV, *bla*CTX-M-1, and *bla*CTX-M-2) were detected by multiplex PCR using specific primers as described in Table 6 [48]. Amplification reactions were set in a 25-μL volume containing 12.5 μL of PCR Master Mix (Thermo Scientific, Waltham, Massachusetts, USA), 1.0 μL (10 pmol) of each of the forward and reverse primers, 1 μL of DNA, and 3.5 μL of nuclease-free water. The multiplex PCR conditions used were as follows: initial denaturation at 95 °C for 5 min, followed by 25 cycles of denaturation at 95 °C for 30 s, annealing at 60 °C for 1 min, and extension at 72 °C for 1 min, with a final extension at 72 °C for 10 min. Appropriate positive and negative controls (sterile phosphate buffer saline) were included in each PCR run. The PCR products were visualized by electrophoresis on a 1.5% agarose gel containing ethidium bromide. The DNA bands were photographed using a UV transilluminator.

### 4.7. Statistical Analysis

Descriptive statistics were used to compute the prevalence of *E. coli* and resistance percentage. The *Z*-test for proportions was done to find out the significant difference in the frequencies of *E. coli* and their resistance percentage among supershops, sampling area, chicken types, production types, meat types, etc. If any of the expected cell frequencies was less than five, Fisher’s exact tests were used. The level of significance was set at *p* < 0.05. SPSS version 22.0 software (IBM Corp., Armonk, N.Y., USA) was used for the analyses.

## 5. Conclusions

The presence of ESBL-producing *E. coli* in frozen chicken meat in Bangladesh poses a risk to human health. Our data indicate the presence of MDR and pXDR ESBL-producing *E. coli* in frozen chicken meat, to which people are regularly exposed, and it warrants the importance of immediate steps being taken to ensure good production and processing practices by the producers, as well as food processors, thus minimizing the contamination of frozen chicken meat in Bangladesh. Furthermore, frozen chicken meat should be properly handled and thoroughly cooked in order to make sure that safe products are consumed. Continuous monitoring and public health efforts targeting food safety management are warranted to proactively manage risks associated with the presence and spread of these antimicrobial-resistant *E. coli* in frozen chicken meat consumed by humans.

## Figures and Tables

**Table 1:** Demographic information of nine branded supershops in five megacities.

Name of Supershops (*N*)	Source of Chicken (%)	Processing of Chicken		Packaging of Chicken	
		Inside Shop *N* (%)	Outside Shop *N* (%)	Inside Shop *N* (%)	Outside Shop *N* (%)
Brand 1 (7)	Contract farm (100)	1 (14.3)	6 (85.7)	6 (85.7)	1 (14.3)
Brand 2 (15)	Contract farm (100)	2 (13.3)	13 (86.7)	10 (66.7)	5 (33.3)
Brand 3 (10)	Contract farm (100)	2 (20.0)	8 (80.0)	8 (80.0)	2 (20.0)
Brand 4 (3)	Contract farm (100)	0	3 (100.0)	2 (66.7)	1 (33.3)
Brand 5 (1)	Contract farm (100)	1 (100.0)	0	1 (100.0)	0
Brand 6 (1)	Contract farm (100)	0	1 (100.0)	1 (100.0)	0
Brand 7 (1)	Contract farm (100)	0	1 (100.0)	0	1 (100.0)
Brand 8 (1)	Contract farm (100)	0	1 (100.0)	1 (100.0)	0
Brand 9 (1)	Contract farm (100)	0	1 (100.0)	1 (100.0)	0

Contract farms: Farmers have the contract with the company (supershop authority) that the company provides the chickens, the feed, veterinary care, and technical advice, while the poultry farmers provide the day-to-day care of the birds, land, and housing, as well as utilities/maintenance of the housing.

**Table 2:** Prevalence of extended-spectrum β-lactamase-producing *Escherichia coli* (ESBL-Ec) and non-ESBL-Ec isolated from frozen chicken meat in different supershops.

Name of Supershops	Total No. of Samples	No. of *E. coli*-Positive Isolates (%)	ESBL-Ec No. (%)	Non-ESBL-Ec No. (%)
Brand 1	23	21 (91.3)	21 (100.0) ^a^	0
Brand 2	40	30 (75.0)	21 (70.0) ^b^	9 (30.0) ^b^
Brand 3	28	24 (85.8)	24 (100.0) ^a^	0
Brand 4	8	3 (37.5)	2 (66.7) ^b^	1 (33.3) ^b^
Brand 5	2	2 (100.0)	2 (100.0) ^a,b^	0
Brand 6	2	2 (100.0)	1 (50.0) ^b^	1 (50.0) ^b^
Brand 7	5	3 (60.0)	3 (100.0) ^a,b^	0
Brand 8	3	1 (33.3)	0	1 (100.0) ^a^
Brand 9	2	0	-	-
**Total**	**113**	**86 (76.1)**	**74 (86.0)**	**12 (14.0)**

ESBL-Ec = ESBL-producing *E. coli*; non-ESBL-Ec = ESBL- non producing *E. coli*; ^a,b^ values in the same column with different superscripts differ significantly (*p* ≤ 0.05).

**Table 3:** Distribution of ESBL-Ec and non-ESBL-Ec isolated from frozen chicken meat.

Variables (*N*)	No. of *E. coli*-Positive Isolates (%)	ESBL-Ec No. (%)	Non-ESBL-Ec No. (%)
**Divisions**			
Dhaka (82)	65 (79.3)	60 (92.3) ^a^	5 (7.7) ^a^
Chattogram (10)	10 (100.0)	10 (100.0) ^a^	0
Sylhet (11)	5 (45.5)	0	5 (100.0) ^b^
Mymensingh (5)	3 (60.0)	3 (100.0) ^a,b^	0
Rajshahi (5)	3 (60.0)	1 (33.3) ^b^	2 (66.7) ^b^
**Chicken types**			
Broiler (82)	63 (76.8)	55 (87.3) ^a^	8 (12.7) ^a^
Cockerel (31)	23 (74.2)	19 (82.6) ^a^	4 (17.4) ^a^
**Production types**			
Organic (10)	5 (50.0)	4 (80.0) ^a^	1 (20.0) ^a^
Non-organic (103)	81 (78.6)	70 (86.4) ^a^	11 (13.6) ^a^
**Meat sample types**			
Breast (27)	22 (81.5)	18 (81.8) ^a^	4 (18.2) ^a^
Drumstick (30)	22 (73.3)	20 (90.9) ^a^	2 (9.1) ^a^
Leg (3)	3 (100.0)	3 (100.0) ^a^	0
Wing (19)	16 (84.2)	14 (87.5) ^a^	2 (12.5) ^a^
Whole-chicken pool sample (34)	23 (67.6)	19 (82.6) ^a^	4 (17.4) ^a^
**Total (113)**	**86 (76.1)**	**74 (86.0)**	**12 (14.0)**

ESBL-Ec = ESBL-producing *E. coli*; non-ESBL-Ec = ESBL-non-producing *E. coli*; ^a,b^ values in the same column with different superscripts differ significantly (*p* ≤ 0.05).

**Table 4:** Supershop-wise distribution of resistant *E. coli* isolated from frozen chicken meat.

Name of Supershops (*N*)	No. (%) of Isolates Resistant to Antimicrobial Agents				
	4–8	9–13	14–18	19–23	24–28
Brand 1 (21)	0	3 (14.3) ^a^	7 (33.3) ^a^	9 (42.9) ^a^	2 (9.5) ^a^
Brand 2 (30)	8 (26.7) ^a^	9 (30.0) ^a,b^	5 (16.7) ^b^	5 (16.7) ^b^	3 (10.0) ^a^
Brand 3 (24)	2 (8.3) ^b^	5 (20.8) ^a,b^	3 (12.5) ^b^	7 (29.2) ^a,b^	7 (29.2) ^b^
Brand 4 (3)	1 (33.3) ^a^	0	1 (33.3) ^a^	1 (33.3) ^a^	0
Brand 5 (2)	0	1 (50.0) ^a,b^	0	0	1 (50.0) ^c^
Brand 6 (2)	0	0	2 (100.0) ^c^	0	0
Brand 7 (3)	0	0	1 (33.3) ^a^	0	2 (66.7) ^c^
Brand 8 (1)	0	1 (100.0) ^b^	0	0	0
**Total**	**11 (12.8)**	**19 (22.1)**	**19 (22.1)**	**22 (25.6)**	**15 (17.4)**

^a,b,c^ Values in the same column with different superscripts differ significantly (*p* ≤ 0.05).

**Table 5:** Prevalence of ESBL-Ec and non-ESBL-Ec genotypes isolated from frozen chicken meat.

Genotypes	ESBL-Ec (*n* = 74)	non-ESBL-Ec (*n* = 12)	Total (*n* = 86)
*bla*TEM	74 (100.0)	12 (100.0)	86 (100.0)
*bla*SHV	0	1 (8.3)	1 (1.2)
*bla*CTX-M-1	0	0	0
*bla*CTX-M-2	0	1 (8.3)	1 (1.2)

ESBL-Ec = ESBL-producing *E. coli*; non-ESBL-Ec = ESBL-non producing *E. coli.*

**Table 6:** Oligonucleotide primers used for the detection of ESBL-encoding genes.

Gene	Name of Primers	Sequence 5′→3′	Amplified Product (bp)
*bla*TEM	TEM-410F TEM-781R	GGTCGCCGCATACACTATTCTC TTTATCCGCCTCCATCCAGTC	372
*bla*SHV	SHV-287F SHV-517R	CCAGCAGGATCTGGTGGACTAC CCGGGAAGCGCCTCAT	231
*bla*CTX-M-1	ctxm1-115F ctxm1-702R	GAATTAGAGCGGCAGTCGGG CACAACCCAGGAAGCAGGC	588
*bla*CTX-M-2	ctxm2-39F ctxm2-145R	GATGGCGACGCTACCCC CAAGCCGACCTCCCGAAC	107

## Supplementary Materials

The following are available online at https://www.mdpi.com/2076-0817/9/6/420/s1: Table S1. Antimicrobial susceptibility pattern of ESBL-Ec and non-ESBL-Ec isolated from frozen chicken meat.
