# Supplementary material for: Antimicrobial Resistance Pattern of Escherichia coli Isolated from Frozen Chicken Meat in Bangladesh
Source: Pathogens. 2020 May 28;9(6):420. doi: 10.3390/pathogens9060420 (PMC7350304; doi:10.3390/pathogens9060420)
Supplement: Supplementary file 1 [file pathogens-09-00420-s001.pdf]

Supplementary Table S1. Antimicrobial susceptibility pattern of ESBL-Ec and Non-ESBL–Ec isolated from frozen chicken meat.

**Antimicrobial category**	**No. of isolates (%)**								
	**Overall (n = 86)**			**ESBL-Ec (n = 74)**			**Non-ESBL-Ec (n = 12)**		
	**R**	**I**	**S**	**R**	**I**	**S**	**R**	**I**	**S**
**Fluoroquinolones**									
Ciprofloxacin	38 (44.2)	20 (23.3)	28 (32.6)	35 (47.3)[a]	17 (23.0)	22 (29.7)	3 (25.0)[b]	3 (25.0)	6 (50.0)
Nalidixic acid	53 (61.6)	15 (17.4)	18 (20.9)	46 (62.2)[a]	14 (18.9)	14 (18.9)	7 (58.3)[a]	1 (8.3)	4 (33.3)
Levofloxacin	29 (33.7)	21 (24.4)	36 (41.9)	27 (36.5)[a]	17 (23.0)	30 (40.5)	2 (16.7)[b]	4 (33.3)	6 (50.0)
Norfloxacin	37 (43.0)	11 (12.8)	38 (44.2)	33 (44.6)[a]	9 (12.2)	32 (43.2)	4 (33.3)[a]	2 (16.7)	6 (50.0)
Gatifloxacin	43 (50.0)	14 (16.3)	29 (33.7)	38 (51.4)[a]	11 (14.9)	25 (33.8)	5 (41.7)[a]	3 (25.0)	4 (33.3)
Pefloxacin	76 (88.4)	0	10 (11.6)	65 (87.8)[a]	0	9 (12.2)	11 (91.7)[a]	0	1 (8.3)
Ofloxacin	49 (57.0)	9 (10.5)	28 (32.6)	43 (58.1)[a]	9 (12.2)	22 (29.7)	6 (50.0)[a]	0	6 (50.0)
**Non-extended spectrum cephalosporins**									
**1st generation**									
Cefalexin	40 (46.5)	0	46 (53.5)	39 (52.7)[a]	0	35 (47.3)	1 (8.3)[b]	0	11 (91.7)
Cephradine	43 (50.0)	0	43 (50.0)	41 (55.4)[a]	0	33 (44.6)	2 (16.7)[b]	0	10 (83.3)
**2nd generation**									
Cefuroxime	37 (43.0)	4 (4.7)	45 (52.3)	36 (48.6)[a]	4 (5.4)	34 (45.9)	1 (8.3)[a]	0	11 (91.7)
Cefaclor	13 (15.1)	38 (44.2)	35 (40.7)	12 (16.2)[a]	31 (41.9)	31 (41.9)	1 (8.3)[a]	7 (58.3)	4 (33.3)
**Extended-spectrum cephalosporins**									
**3rd generation**									
Cefixime	0	4 (4.7)	82 (95.3)	0	2 (2.7)	72 (97.3)	0	2 (16.7)	10 (83.3)
Ceftazidime	25 (29.1)	3 (3.5)	58 (67.4)	25 (33.8)	3 (4.1)	46 (62.2)	0	0	12 (100)
Ceftriaxone	2 (2.3)	9 (10.5)	75 (87.2)	2 (2.7)	9 (12.2)	63 (85.1)	0	0	12 (100)
Cefotaxime	46 (53.5)	12 (14.0)	28 (32.6)	46 (62.2)	12 (16.2)	16 (21.6)	0	0	12 (100)
**4th generation**									
Cefepime	62 (72.1)	13 (15.1)	11 (12.8)	60 (81.1)[a]	10 (13.5)	4 (5.4)	2 (16.7)[b]	3 (25.0)	7 (58.3)
**Cephamycins**									
Cefoxitin	42 (48.8)	8 (9.3)	36 (41.9)	41 (55.4)[a]	7 (9.5)	26 (35.1)	1 (8.3)[a]	1 (8.3)	10 (83.3)
**Penicillins**									
Ampicillin	77 (89.5)	5 (5.8)	4 (4.7)	66 (89.2)[a]	5 (6.8)	3 (4.1)	11 (91.7)[a]	0	1 (8.3)
Amoxicillin	79 (91.9)	0	7 (8.1)	68 (91.9)[a]	0	6 (8.1)	11 (91.7)[a]	0	1 (8.3)
**Penicillins + β-lactamase inhibitors**									
Amoxicillin-clavulanic acid	36 (41.9)	28 (32.6)	22 (25.6)	31 (41.9)[a]	25 (33.8)	18 (24.3)	5 (41.7)[a]	3 (25.0)	4 (33.3)
**Antipseudomonal penicillins + β-lactamase inhibitors**									
Piperacillin-tazobactam	61 (70.9)	23 (26.7)	2 (2.3)	54 (73.0)[a]	18 (24.3)	2 (2.7)	7 (58.3)[a]	5 (41.7)	0
**Carbapenems**									

Imipenem	41 (47.7)	19 (22.1)	26 (30.2)	40 (54.1)[a]	15 (20.3)	19 (25.7)	1 (8.3)[b]	4 (33.3)	7 (58.3)
Meropenem	36 (41.9)	14 (16.3)	36 (41.9)	36 (48.6)	13 (17.6)	25 (33.8)	0	1 (8.3)	11 (91.7)
**Polymyxins**									
Colistin	9 (10.5)	0	77 (89.5)	9 (12.2)	0	65 (87.8)	0	0	12 (100)
Polymyxin B	6 (7.0)	0	80 (93.0)	6 (8.1)	0	68 (91.9)	0	0	12 (100)
**Monobactams**									
Aztreonam	1 (1.2)	5 (5.8)	80 (93.0)	1 (1.4)	5 (6.8)	68 (91.9)	0	0	12 (100)
**Aminoglycosides**									
Gentamicin	7 (8.1)	7 (8.1)	72 (83.7)	6 (8.1)[a]	6 (8.1)	62 (83.8)	1 (8.3)[a]	1 (8.3)	10 (83.3)
Tobramycin	7 (8.1)	13 (15.1)	66 (76.7)	5 (6.8)[a]	13 (17.6)	56 (75.7)	2 (16.7)[b]	0	10 (83.3)
Amikacin	15 (17.4)	43 (50.0)	28 (32.6)	14 (18.9)[a]	38 (51.4)	22 (29.7)	1 (8.3)[b]	5 (41.7)	6 (50.0)
Streptomycin	50 (58.1)	17 (19.8)	19 (22.1)	47 (63.5)[a]	17 (23.0)	10 (13.5)	3 (25.0)[b]	0	9 (75.0)
Neomycin	24 (27.9)	41 (47.7)	21 (24.4)	22 (29.7)[a]	39 (52.7)	13 (17.6)	2 (16.7)[a]	2 (16.7)	8 (66.7)
**Tetracyclines**									
Tetracycline	73 (84.9)	5 (5.8)	8 (9.3)	62 (83.8)[a]	5 (6.8)	7 (9.5)	11 (91.7)[a]	0	1 (8.3)
Oxytetracycline	80 (93.0)	0	6 (7.0)	68 (91.9)[a]	0	6 (8.1)	12 (100.0)[a]	0	0
Doxycycline	59 (68.6)	20 (23.3)	7 (8.1)	52 (70.3)[a]	16 (21.6)	6 (8.1)	7 (58.3)[a]	4 (33.3)	1 (8.3)
**Folate pathway inhibitors**									
Trimethoprim-sulphamethoxazole	76 (88.4)	0	10 (11.6)	66 (89.2)[a]	0	8 (10.8)	10 (83.3)[a]	0	2 (16.7)
**Glycylcyclines**									
Tigecycline	2 (2.3)	15 (17.4)	69 (80.2)	1 (1.4)[a]	13 (17.6)	60 (81.1)	1 (8.3)[b]	2 (16.7)	9 (75.0)
**Phenicols**									
Chloramphenicol	27 (31.4)	0	59 (68.6)	21 (28.4)[a]	0	53 (71.6)	6 (50.0)[b]	0	6 (50.0)
**Macrolids**									
Azithromycin	30 (34.9)	0	56 (65.1)	29 (39.2)[a]	0	45 (60.8)	1 (8.3)[b]	0	11 (91.7)

n = number of isolates, R = Resistant, I = Intermediate, S = Susceptible; ESBL-Ec = ESBL-producing *E. coli*; Non-ESBL-Ec = ESBL- non producing *E. coli*; [a,b]Values in the same row with different superscripts differ significantly (p <0.05).
